# Supplementary material for: Sequentially amplified circularly polarized ultraviolet luminescence for enantioselective photopolymerization
Source: Nat Commun. 2020 Nov 9;11:5659. doi: 10.1038/s41467-020-19479-1 (PMC7652877; doi:10.1038/s41467-020-19479-1)
Supplement: Supplementary file 1 — Supplementary Information [file 41467_2020_19479_MOESM1_ESM.pdf]

Supplementary Information for

Sequentially amplified circularly polarized ultraviolet luminescence for enantioselective photopolymerization

Dongxue Han,[1, 2] *Xuefeng Yang,*[2, 3] *Jianlei Han,*[2] *Jin Zhou,*[2] *Tifeng Jiao,*[1] *Pengfei Duan* *[2, 3]

1 State Key Laboratory of Metastable Materials Science and Technology, Yanshan University, No. 438 West Hebei Street, Qinhuangdao 066004, P. R. China.

2 CAS Center for Excellence in Nanoscience, CAS Key Laboratory of Nanosystem and Hierarchical Fabrication, National Center for Nanoscience and Technology (NCNST), No. 11 ZhongGuanCun BeiYiTiao, Beijing 100190, P. R. China.

3 University of Chinese Academy of Sciences, No.19(A) Yuquan Road, Shijingshan District, Beijing 100049 (China).

Supplementary Methods

Synthesis of *R*-/*S*-TP. A mixture of *R*(*S*)-4,12-dibromo[2,2]paracyclophane (200 mg, 0.546 mmol), 1,4-biphenylboronic acid (270 mg, 1.363 mmol), [$Pd(PPh_3)_4$] (75 mg, 0.065 mmol), aqueous K_2CO_3 (2 M, 15 mL) and toluene (35 mL) was heated to 90℃ refluxed overnight under N_2 . After cooling to RT, the reaction mixture was poured into 100 mL of water and extracted with CH_2Cl_2. The organic layers were washed with water and dried over $MgSO_4$ and the volatiles were removed under vacuum. The residue was purified by column chromatography on silica (petroleum ether/CH_2Cl_2 = 10/1) to give as a white solid (100 mg, 35.7%). ***S*-TP**: ^{1}H-NMR (400 MHz, DMSO-d_6) δ: 7.8 (s, 2H), 7.51-7.52 (m, 8H), 7.41(d, 2H), 7.3 (d, 2H), 7.25 (m, 8H), 7.01 (d, 2H), 2.88 (s, 8H); MS (MALDI) *m*/*z*: calcd: 512.7, found: 512.1$[M]^+$. ***R*-TP**: ^{1}H-NMR (400 MHz, DMSO-d_6) δ: 7.8 (s, 2H), 7.51-7.54 (m, 8H), 7.41(d, 2H), 7.3 (d, 2H), 7.26 (m, 8H), 7.02 (d, 2H), 2.89 (s, 8H). *m*/*z*: calcd: 512.7, found: 512.3$[M]^+$.

Determination of TTA-UC quantum yield by relative method. The upconverted emission quantum efficiency (Φ_{UC}) was determined relative to a standard according to the following equation

$$\Phi_{\mathrm{UC}} = 2\Phi_{\mathrm{std}}\left(\frac{A_{\mathrm{std}}}{A_{\mathrm{uc}}}\right)\left(\frac{I_{\mathrm{uc}}}{I_{\mathrm{std}}}\right)\left(\frac{\eta_{\mathrm{uc}}}{\eta_{\mathrm{std}}}\right)^2 \quad (1)$$

Where Φ, A, I and η represent a quantum yield, absorbance, integrated photoluminescence spectral profile, and refractive index of the solvents used as a standard, respectively. The subscripts UC and std denote the parameters of the tested upconversion and standard systems, respectively. The UC quantum yield was determined relative to a standard, Coumarin 6 in DMF (Φ_{std} = 72%) and Coumarin 6 in liquid crystal 5CB (Φ_{std} = 63%) under 445 nm excitation. Note that the theoretical maximum of Φ_{UC} is standardized to be 1 (100%).

Supplementary Figures

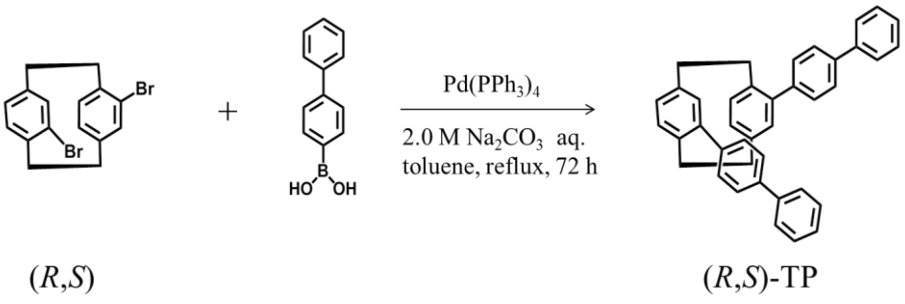

Supplementary Figure 1. Synthesis route of (*R*,*S*)-4,12-biphenyl[2,2]paracyclophane (*R*-/*S*-TP)

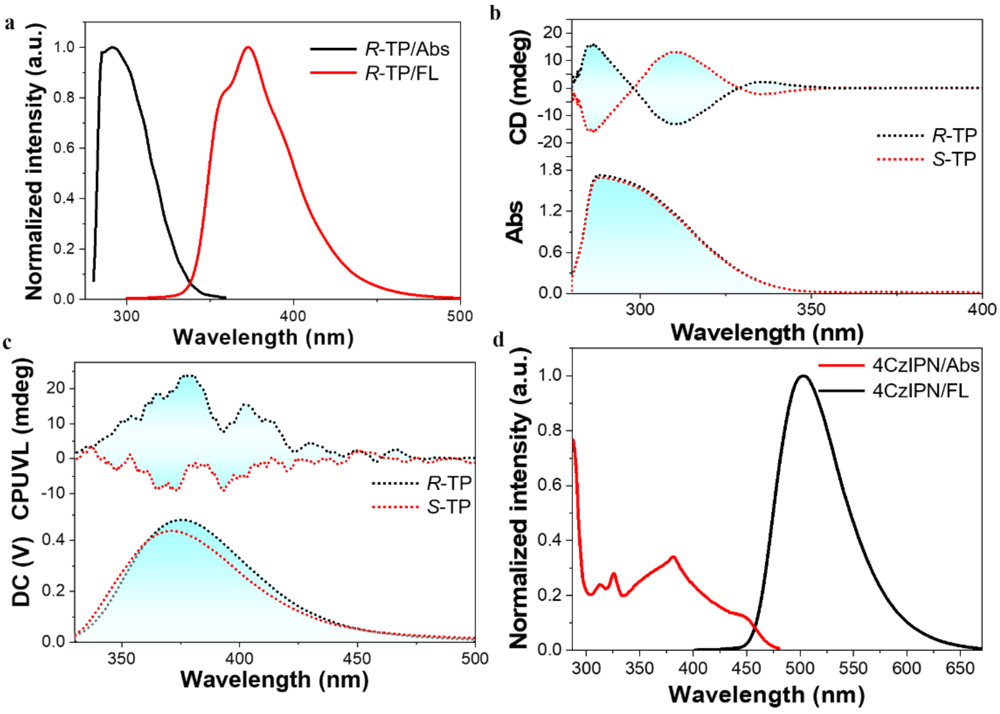

Supplementary Figure 2. Optical properties of *R*-/*S*-TP and 4CzIPN in toluene solution. (**a**) Normalized absorption and emission spectra of *R*-TP (0.01 mM, λ_{ex} = 290 nm) in toluene solution. (**b**) CD spectra of *R*(*S*)-TP in toluene solution ([*R*-TP] = [*S*-TP] = 0.01 mM). (**c**) CPL spectra of *R*(*S*)-TP in toluene solution ([*R*-TP] = [*S*-TP] = 0.01 mM, λ_{ex} = 290 nm). (**d**) Normalized absorption and emission spectra of 4CzIPN (0.01 mM, λ_{ex} = 445 nm) in toluene solution.

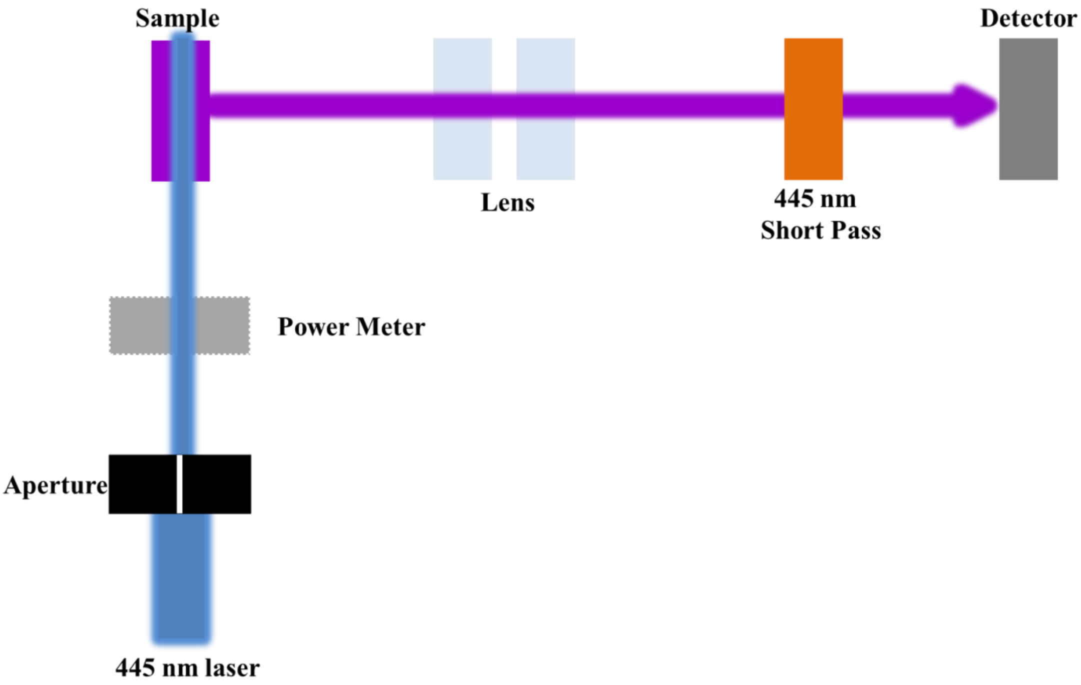

Supplementary Figure 3. Schematic illustration of the experimental setup for characterization of TTA-UC emission.

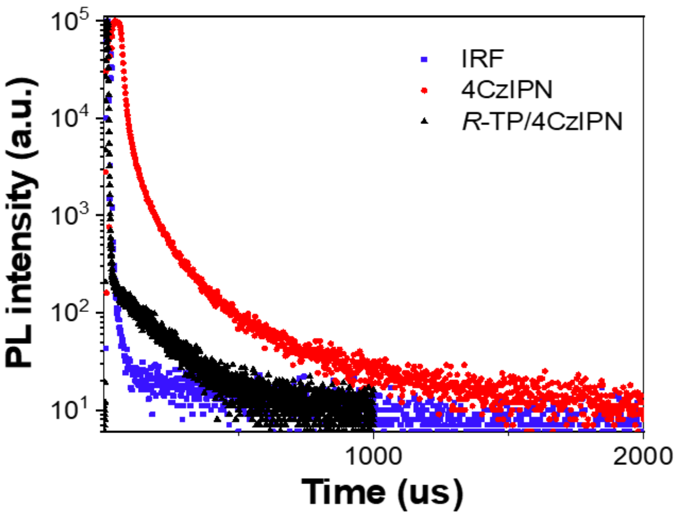

Supplementary Figure 4. Time-resolved emission of *R*-TP and 4CzIPN. Photoluminescence (PL) decay of the deaerated toluene solution of 4CzIPN (0.1 mM) (red line) and the mixture of *R*-TP/4CzIPN ([*R*-TP] = 3 mM, [4CzIPN] = 0.1 mM) (black line) at 500 nm under pulsed excitation of 445 nm CW laser (A 445 nm short-pass was used).

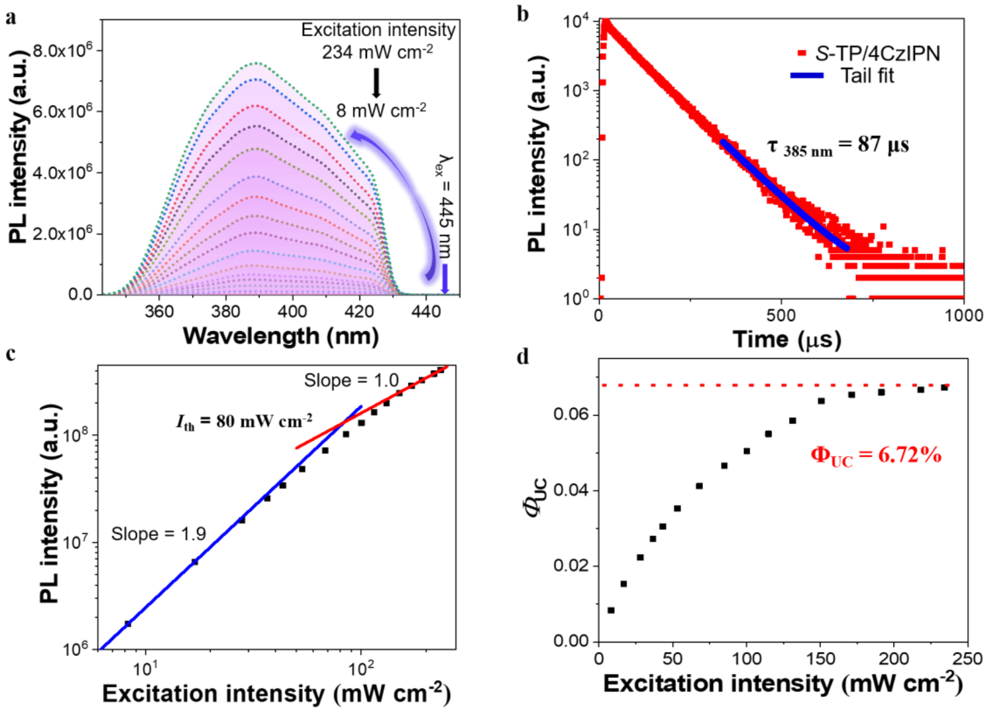

Supplementary Figure 5. Upconversion process of *S*-TP/4CzIPN in deaerated toluene solution. (**a**) Upconversion emission spectra of *S*-TP/4CzIPN with different incident excitation intensities of the 445 nm CW laser in deaerated toluene solution. (**b**) Time-resolved upconverted emission at 385 nm of the *S*-TP/4CzIPN in toluene solution (λ_{ex} = 445 nm). (**c**) Double-logarithmic plots of the Upconversion emission intensity of *S*-TP/4CzIPN in deaerated toluene solution as a function of the excitation intensity. (**d**) Upconversion efficiencies of *S*-TP/4CzIPN in deaerated toluene solution with different excitation light intensities of the 445 nm CW laser, ([*S*-TP] = 3 mM, [4CzIPN] = 0.1 mM, a 445nm short-pass filter was used).

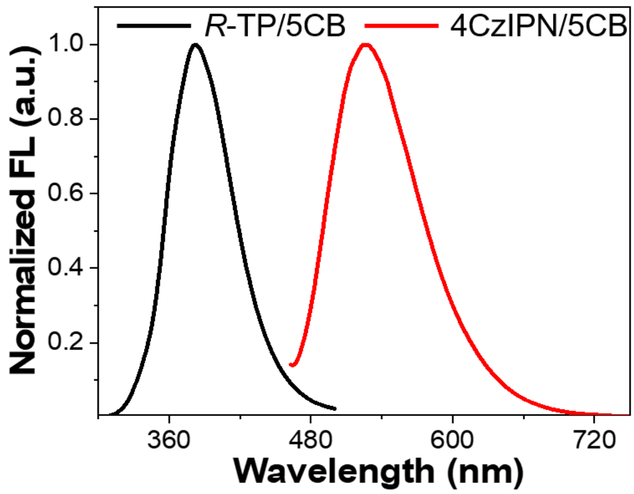

Supplementary Figure 6. Normalized emission spectra of *R*-TP and 4CzIPN in 5CB. The weight ratios of *R*-TP (λ_{ex} = 290 nm) and 4CzIPN (λ_{ex} = 445 nm) were 10 wt% and 3 mol%, respectively.

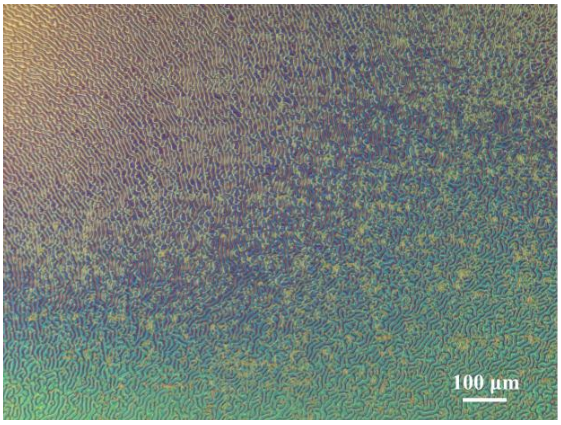

Supplementary Figure 7. POM image of three components system *R*-TP/4CzIPN/5CB. The weight ratios of *R*-TP/5CB and 4CzIPN/*R*-TP were 10 wt% and 3 mol%, respectively.

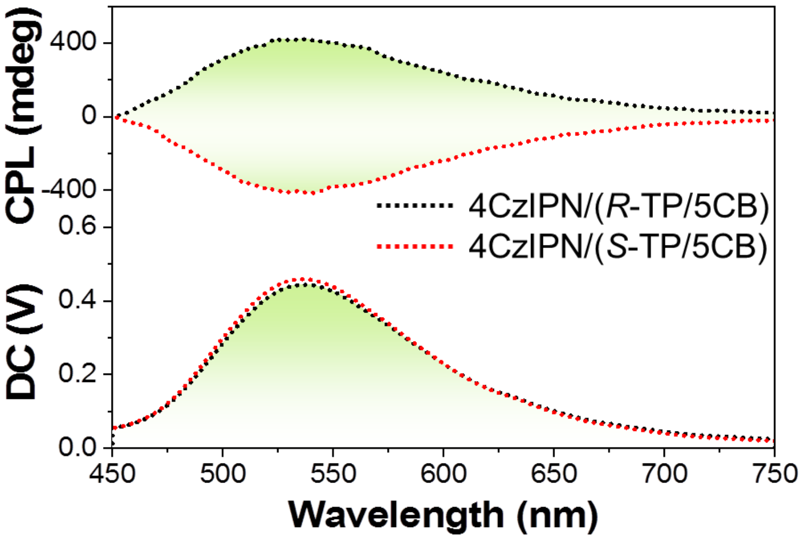

Supplementary Figure 8. CPL spectra of 4CzIPN in N*LC. The weight ratio of *R*(*S*)-TP/5CB was 10 wt% (λ_{ex} = 445 nm).

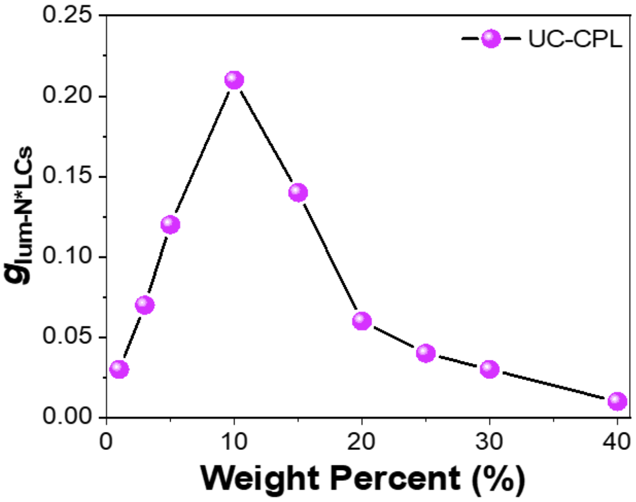

Supplementary Figure 9. The tendency of UC-CPL dissymmetry factor g_{lum} with different weight ratios of acceptor in the liquid crystal. λ_{ex} = 445 nm.

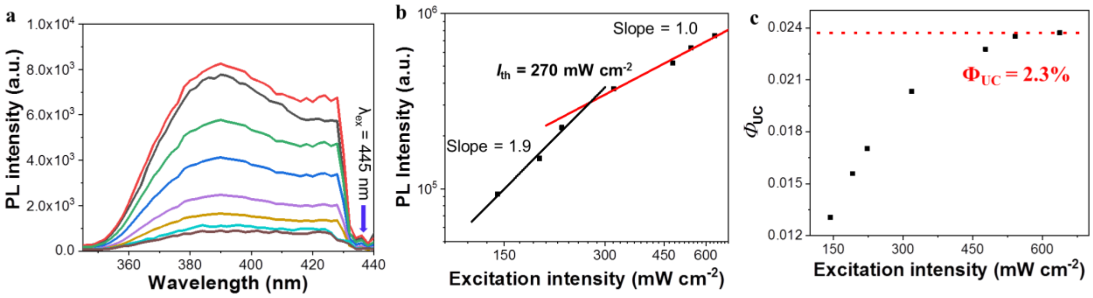

Supplementary Figure 10. The upconverted emission of *R*-TP/4CzIPN in 5CB. a) Upconversion emission spectra of *R*-TP/4CzIPN with different incident excitation intensities of the 445 nm CW laser in a room-temperature nematic liquid crystal (5CB); b) Double-logarithmic plots of the upconversion emission intensity of *R*-TP/4CzIPN in 5CB as a function of the excitation intensity. c) Upconversion efficiencies of *R*-TP/4CzIPN in 5CB with different excitation light intensities of the 445 nm CW laser. For all the upconversion measurements, a 445 nm short-pass filter was used.

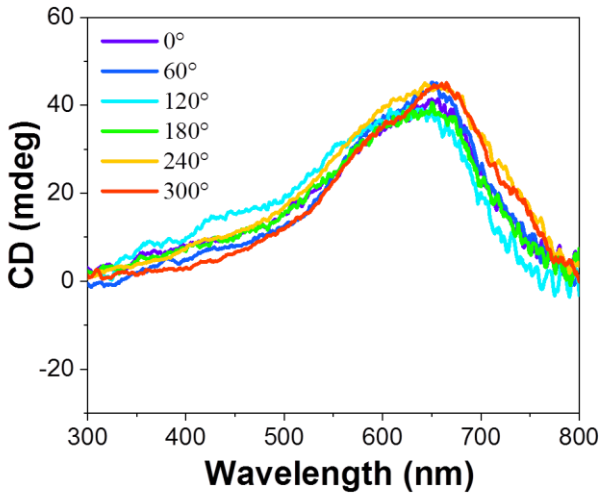

Supplementary Figure 11. The CD spectra at various rotation angles about surface normal for the PDA film. The UC-CPUVL generated from the excited *R*-TP/4CzIPN in N*LC with incident excitation of the 445 nm CW laser (A 445 nm short-pass was used).
